# The Effectiveness of Cognitive Behavioral Therapy on Depression and Sleep Problems for Climacteric Women: A Systematic Review and Meta-Analysis

**DOI:** 10.3390/jcm13020412

**Published:** 2024-01-11

**Authors:** Ji-Hyun Kim, Hea-Jin Yu

**Affiliations:** College of Nursing, Sahmyook University, Seoul 01795, Republic of Korea

**Keywords:** climacteric, menopause, depressive symptoms, sleep-related problem, sleep quality, cognitive behavioral therapy

## Abstract

(1) Background: Women in their middle years undergoing perimenopause encounter a range of physical and psychological alterations attributed to hormonal changes. The prominent symptoms among menopausal women are depressive symptoms and sleep-related problems. The aim of this study was to conduct a meta-analysis examining the effects of Cognitive Behavioral Therapy (CBT) on women going through menopause, specifically focusing on depressive symptoms and sleep problems. We analyzed studies conducted both within the country and across international settings over the last decade. (2) Methods: A search of the literature was conducted—a targeted search, exclusively considering randomized controlled trials (RCTs) that were published within the timeframe spanning from 15 June 2013 to 15 June 2023. (3) Findings: Upon reviewing nine studies that satisfied our inclusion criteria and involved a total of 923 participants, it was noted that four of these studies incorporated diverse cognitive-behavioral strategies. Among the nine studies, a total of four were included in the meta-analysis: two measured depressive symptoms, and two measured sleep quality. The combined effect size for depressive symptoms was found to be 3.55 (95% confidence interval: −5.48, −1.61; *p* < 0.05), and for sleep quality, it was 0.78 (95% confidence interval: −1.32, −0.25; *p* = 0.004). (4) Conclusions: Our review emphasizes the necessity for conducting larger-scale studies focused on the application of CBT for women experiencing menopausal symptoms. Additionally, it is recommended to approach the interpretation of these results with caution due to discrepancies in methodology and the overall quality of the studies. Further clinical trials are necessary to establish the ideal number of CBT sessions needed for the effective treatment of depression in menopausal women. Future studies should cover a wider range of geographical locations, including more countries, and focus on various outcomes such as depressive symptoms and sleep quality.

## 1. Introduction

On average, women are expected to live approximately 85 years, while men have a reported average life expectancy of around 79 years. This suggests that, on average, women tend to live about 6 years longer than men [1]. Consequently, women typically live through approximately one-third of their lifetime in the post-middle-age phase. Menopause refers to the natural aging process in which ovarian hormone production gradually decreases, leading to the complete cessation of menstruation. This transition typically begins in the late 40s and progresses [2]. Perimenopause refers to the years leading up to and following menopause, during which ovarian function gradually diminishes. Menopause occurs during the perimenopausal phase, defined as a specific instance occurring 12 consecutive months after a woman experiences her last menstrual period. The interval leading up to this event is marked by potential shifts in monthly menstrual cycles, the occurrence of hot flashes, or the emergence of other symptoms [2,3].

Most of the time, middle-aged women going through the perimenopause/climacteric phase experience various physical and psychological changes due to hormonal fluctuations. Some common physical changes include decreased skin elasticity, decreased libido, palpitations, hot flashes, and facial flushing. For non-disabled women, entering their 40s and experiencing irregular menstruation can be considered the onset of menopause. In some cases, menopause may occur earlier in disabled women compared to non-disabled women [3].

It is a well-known fact that the primary causes of menopause are the decline in ovarian function and a reduction in female hormones. As menopause approaches, ovarian function declines rapidly, leading to a decrease in the secretion of the female hormone estrogen. Estrogen influences various parts of the female body, including the uterus, breasts, brain, heart, blood vessels, and bones. A reduction in estrogen affects these diverse organs, leading to the onset of menopausal symptoms. Factors such as family history, smoking, irregular lifestyle habits, stress, the course of disease treatment, and chronic conditions like hypertension and diabetes can exacerbate ovarian function decline [1,3].

One of the prominent psychological symptoms among menopausal women is depression [4,5]. Menopausal women often experience depressive disorders due to changes in female hormones as well as social role changes, such as their children becoming independent and the death of elderly family members [1]. Symptoms of depression include lethargy, feelings of worthlessness, low self-esteem, weight gain or loss, impaired memory, reduced concentration, and sleep difficulties, all of which have a significant impact on a person’s life and can cause a great deal of distress [6]. Also, depression signifies an emotional state characterized by concerns, feelings of failure, loss, helplessness, and worthlessness, which are the results of negative perceptions about oneself.

During the morning, depressive symptoms escalate, including early awakening, noticeable psychomotor agitation or retardation, substantial anorexia or weight loss, and heightened feelings of guilt. This differentiation is significant because, overall, women are more susceptible to encountering these symptoms than men. Additionally, atypical depressive features, like overeating and oversleeping, are about four times more common in women than in men, generally [7,8]. Depression not only involves a dysphoric emotional state but also encompasses complex mental and physical symptoms such as pain, changes in appetite, suicidal thoughts and attempts, and insomnia [7]. Therefore, depression in menopausal women cannot be overlooked.

Menopausal women may also experience difficulty falling asleep, tossing and turning during sleep, poor sleep quality, fatigue, and daytime sleepiness due to symptoms such as hot flashes and palpitations [9]. It is a well-known fact that sleep disturbances and difficulties have a serious negative impact on both physical and mental health [9]. The prevalence of insomnia in menopausal women is approximately 15%, and persistent sleep disturbances can increase the risk of various cardiovascular diseases, stroke, diabetes, and metabolic syndrome and, in severe cases, it can lead to death. In addition, the inability to sleep prevents restoration from fatigue, resulting in daytime tiredness and drowsiness. Multiple bodily functions weaken, leading to the initiation or exacerbation of various physical ailments. Insufficient sleep also has adverse effects on cognitive functions, including memory and concentration. Prolonged insomnia is reported to potentially progress to depression in approximately 50% of cases. Therefore, there is a close relationship between depression and sleep disturbances [10,11]. Depressive symptoms can include sleep disturbances and, conversely, sleep disturbances can trigger depression, underscoring the importance of early assessment and appropriate management [12].

Cognitive behavioral therapy (CBT) places emphasis on ‘cognition’, meaning our thoughts, which are assumed to significantly influence emotions, behaviors, and interpersonal relationships, with the belief that these elements are closely interconnected. In CBT, the focus on the client’s cognition is used to understand and explain psychological issues, and various techniques are employed to promote changes in cognition. Extensive evidence supports its effectiveness in alleviating symptoms of depression and sleep disturbances. Originally, hormone replacement therapy was considered the most effective treatment for menopausal symptoms. However, many postmenopausal women are reluctant to opt for this method due to the increased risks of breast cancer, blood clots, and heart disease associated with it. [13,14].

Our research study aims to conduct a systematic review and meta-analysis assessing the impact of CBT on women undergoing menopause who are grappling with depressive symptoms and sleep-related problems. The findings of our review are anticipated to furnish fundamental insights into the treatment and clinical management of depression and sleep issues in menopausal women, highlighting the efficacy of non-pharmacological interventions such as CBT.

The specific purposes of this study, which involves conducting a meta-analysis on the effects of CBT applied to perimenopausal women with symptoms of depression and sleep disorders, are as follows:

(1) To establish the overall features of CBT in the search process;
(2) To evaluate the effect size of the impact of CBT in mitigating depressive symptoms;
(3) To measure the effect size of the effect of CBT on issues related to sleep.

## 2. Materials and Methods

### 2.1. Study Design and Procedures

**Figure 1:**
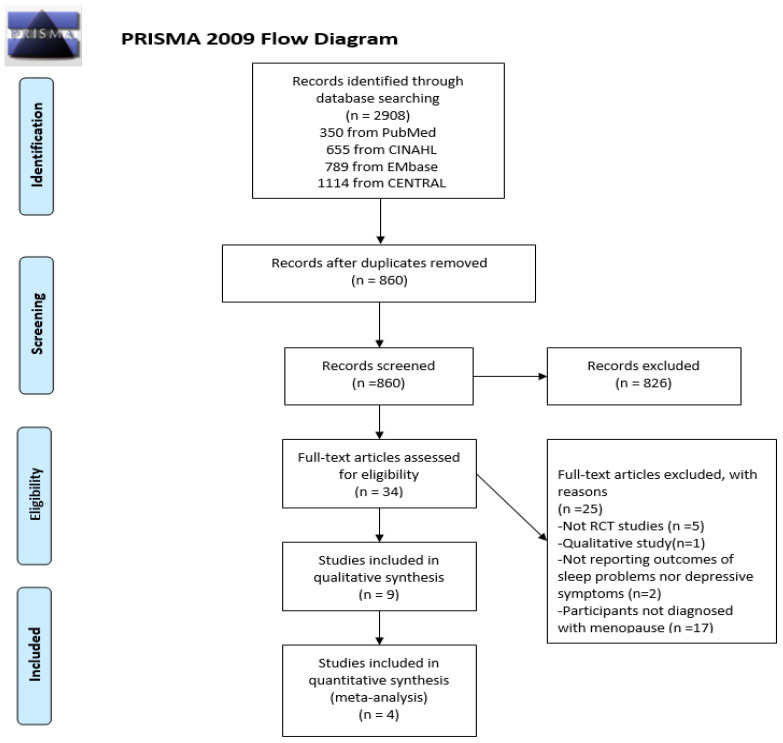
PRISMA flow diagram [13].

The study adhered to the systematic review and meta-analysis protocols outlined in the Preferred Reporting Items for Systematic Review and Meta-analysis (PRISMA) guidelines and the procedures specified in the *Cochrane Handbook for Systematic Reviews of Interventions* [13]. Figure 1 displays a flow chart illustrating the process. The protocol for the review has been registered in INPLASY (International Platform of Registered Systematic Review and Meta-analysis Protocols) with the registration ID INPLASY202410033.

### 2.2. Inclusion Criteria and Criteria of Material Selection

The review encompassed RCTs that satisfied the following conditions: (1) studies concentrating on women in the perimenopausal stage within the age range of 40 to 65 years; (2) studies encompassing assessments related to depression; (3) studies involving assessments related to sleep disorders; (4) RCTs; (5) and studies that were published in the English language.

### 2.3. Search Strategy

To identify relevant and qualified research articles, a comprehensive search was conducted using the following databases: Cumulative Index to Nursing and Allied Health Literature (CINAHL), PubMed, EMbase, and Cochrane Central Register of Controlled Trials (CENTRAL). The search terms included a variety of relevant terms such as “climacteric”, “menopause”, “depression”, “emotional depression”, “depressive symptoms”, “sleep disorder”, “sleep-related problem”, “cognitive behavioral therapy”, “psychological intervention”, “therapy”, “counseling”, and “program.” To enhance the comprehensiveness of the search, the search terms were expanded or “exploded”, incorporating controlled vocabularies in addition to including free-text terms.

### 2.4. Study Selection and Data Extraction

After identifying pertinent studies, JHK and HJY participated in discussions to reconcile any discrepancies. A consistent methodology was employed to systematically collect and organize information regarding the study population and the specifics of CBT in both the intervention and control groups. A Cochrane review data extraction form was utilized to compile trial characteristics. 

### 2.5. Risk of Bias Assessment and Data Analysis

**Figure 2:**
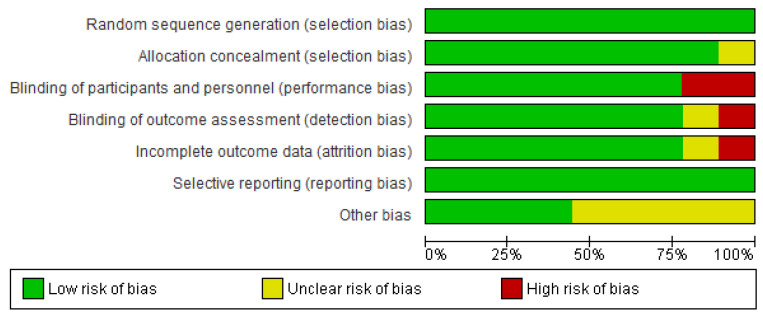

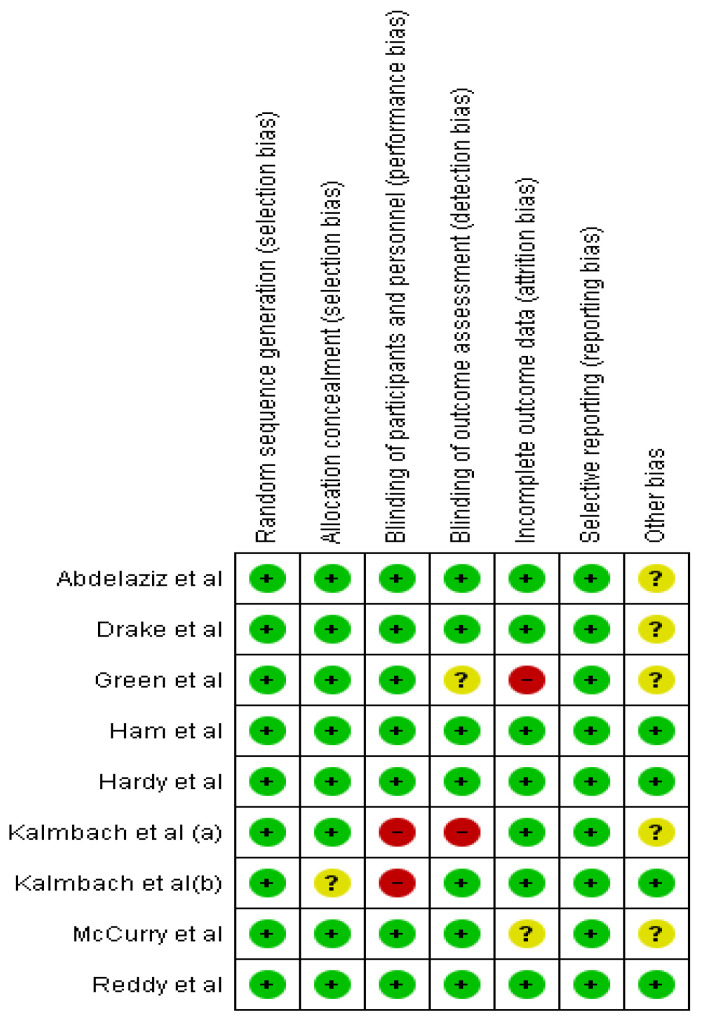
Risk of bias (RoB) [17,18,19,20,21,22,23,24,25].

To assess the quality of each study, we utilized the Cochrane Risk of Bias (RoB) tool, which scrutinizes six specific domains: random sequence generation, allocation concealment, blinding of participants and personnel, blinding of incomplete outcome data, and selective outcome reporting [15]. Each author independently evaluated bias throughout the selection process, and any discrepancies were resolved through discussion (Figure 2). Review Manager 5.3 software was used to determine effect sizes, assessing the impact of psychological therapies on depressive symptoms and sleep quality. The overall effect size was established using Standardized Mean Differences (SMD), 95% confidence intervals, and weighted mean difference calculations. A fixed-effect model was applied due to the homogeneity of the data, indicating a shared real treatment effect. Statistical tests, such as the Q-test and Higgins’ I^2^ Cochran’s statistics, were employed to quantify statistical heterogeneity comprehensively. Forest plots were employed for visual assessment of effect sizes, confidence intervals, and their overlap before applying statistical techniques. Following the methodology outlined by Higgins et al. [16], levels of heterogeneity were categorized as low (25%), moderate (50%), and high (75%). 

All nine Randomized Controlled Trials (RCTs) (100%) provided a sufficient description of their randomization sequences. Allocation concealment was adequately demonstrated in eight out of nine studies (88%), with one study (11%) considered ambiguous due to a lack of a clear description. In two of the nine investigations (22%), it was not feasible to implement blinding for participants, staff, or outcomes. In one study (11%), the blinding of study personnel was considered ambiguous due to insufficient detail. Among the five studies (55%) evaluated for attrition bias, none showed frequent missing data. However, in the remaining four studies (44%), no information was provided regarding either the attrition rate or the reasons for missing data. Finally, none of the studies (100%) assessed for selective reporting demonstrated selective reporting bias.

## 3. Results

Due to the restricted number of randomized controlled trials (RCTs) in our analysis, we were unable to evaluate the existence of publication bias. Usually, tests such as scrutinizing asymmetry in funnel plots and employing Egger’s regression test are utilized when there are ten or more studies in a meta-analysis, as advised by Dalton et al. in 2016 [26]. However, owing to the limited number of trials in our study, all the methods employed to potentially identify publication bias lacked adequate statistical power.

### 3.1. Attributes of the Participants

Table 1 outlines the essential characteristics of the research studies included in our systematic review and meta-analysis. Nine studies, comprising 923 individuals with peri- or post-menopause, met the inclusion criteria. Sample sizes ranged from 44 to 150, with a median of 90. The participants’ mean age varied from 49.16 to 56.44 years, with a median age of 54.09 years.

### 3.2. Features of the Studies

In the last five years, three out of the five studies were published, covering the years 2007 to 2018. The studies were conducted in six different countries: Canada (*n* = 1), Saudi Arabia (*n* = 1), the United States (*n* = 4), India (*n* = 1), the United Kingdom (*n* = 1), and South Korea (*n* = 1). Among the nine studies, five employed individual intervention delivery, while the remaining four utilized web-based and group formats, respectively. Various measures were used to assess depressive symptoms in climacteric women, including the Beck Depression Inventory-II (BDI-II), the Patient Health Questionnaire-9 (PHQ-9), the Geriatric Depression Scale (GDS), the Center for Epidemiologic Studies Depression Scale (CES-D), the 10-item Center for Epidemiologic Studies Depression Scale (CES-D-R), and the Montgomery-Asberg Depression Rating Scale (MADRS). Additionally, sleep-related issues in climacteric women were measured using the Pittsburgh Sleep Quality Index (PSQI), Insomnia Severity Index (ISI), Ford Insomnia Response to Stress Test (FORD), Dysfunctional Beliefs and Attitudes About Sleep Scale, and the Diary-based Daytime Sleepiness scales. The average number of sessions for CBT was 5.5 weeks, with an average of 5.5 sessions and an average duration of 60 min per session. The outcome variables were related to sleep duration and included a total sleep time in four cases, sleep latency in four cases, and wake-up time after falling asleep in four cases. Outcome variables related to sleep quality included sleep efficiency in three cases and self-rated sleep quality in six cases. Outcome variables related to beliefs and attitudes about sleep included the severity of insomnia in four cases and irrational beliefs and attitudes about sleep in one case. Data were collected through subjective sleep diary entries, objective measures such as actigraphy, and comprehensive sleep assessments, although the majority of data was obtained through sleep diary entries, with only a few cases utilizing actigraphy and comprehensive sleep assessments.

### 3.3. Efficacy of the Interventions on Depressive Symptoms and Sleep-Related Issues

**Figure 3:**
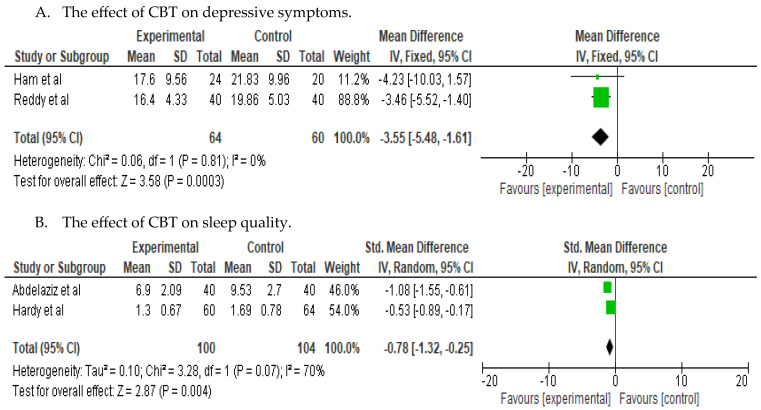
Forest plots of the effect of CBT on depressive symptoms and sleep quality [17,20,21,25].

The efficacy of CBT on depressive symptoms and sleep-related problems is depicted in Figure 3, featuring forest plots illustrating the corresponding effect sizes of CBT. Concerning depressive symptoms, two trials (124 participants) in the review provided data suitable for pooling. When examined individually, two studies revealed statistically significant improvement in the intervention groups regarding depressive symptoms. Using the fixed-effect model, the combined mean difference (MD) in the improvement of depressive symptoms between participants undergoing CBT and those in the control group was statistically significant (d = 3.55; 95% CI, −5.48, −1.61; *p* < 0.05), indicating a substantial effect size. The low heterogeneity was evident from the Cochrane Q and I^2^ scores (Q value = 0.06; *p* = 0.81; I^2^ = 0%). At the 1-month follow-up, depressive symptoms decreased by approximately 15% in 64 participants in the intervention group, while among the remaining 60 participants in the control group, depressive symptoms increased by approximately 16%.

Regarding sleep quality, two of the five studies with 204 participants measured it. Both studies reported an enhancement in sleep quality within the intervention group. At the 6-month follow-up, sleep quality improved by approximately 11.5% in 100 participants in the intervention group and by approximately 7% among the remaining 104 participants in the control group. The Standardized Mean Difference (SMD) of sleep quality was (d = 0.78; 95% CI, −1.32, −0.25; *p* = 0.004), indicating a medium effect size. The Cochran Q test and I^2^ scores for sleep quality (Q value = 3.28, *p* = 0.07, I^2^ = 70%) indicated substantial heterogeneity.

### 3.4. Publication Bias

We could not assess publication bias because there were not enough randomized controlled trials RCTs included in our meta-analysis. Typically, researchers employ tests like funnel plot asymmetry and Egger’s regression test when there are ten or more studies in a meta-analysis, as recommended by Dalton et al. in 2016 [26]. Given the limited number of trials in our study, all methods available for detecting potential publication bias lacked the statistical power necessary for meaningful analysis.

### 3.5. Subgroup Analyses

We conducted subgroup analyses based on the heterogeneity observed in the dependent variable. The analyses were carried out according to the types of scales (whether the Pittsburgh Sleep Quality Index was used), the form of intervention (individual vs. group), and the average number of intervention applications (5.5 times or more vs. less than 5.5 times). In the subgroup analysis based on the type of scale, when measuring the quality of sleep with the Pittsburgh Sleep Quality Index, the effect size was statistically significant at −2.8 (95% CI: −3.71, −2.06), indicating a significantly larger effect compared to measurements with other types of sleep scales (*p* < 0.001, I^2^ = 0%), and the heterogeneity was low. In the subgroup analysis based on the form of intervention, for the quality of sleep, group intervention showed a statistically significant effect size of −2.73 (95% CI: −3.69, −1.78) compared to individual intervention (*p* < 0.001, I^2^ = 0%), and the heterogeneity was low. In the analysis based on the number of intervention applications, when the average application frequency was 5.5 times or more, the effect size was −0.88 (95% CI: −1.27, −0.48), which was statistically significant (*p* < 0.001, I^2^ = 27%), and the heterogeneity was low. However, when the intervention application frequency was less than 5.5 times, statistical significance was achieved (*p* < 0.009, I^2^ = 62%), but the heterogeneity was moderate. Overall, the findings suggest that the type of scale, form of intervention, and the number of intervention applications may influence the effectiveness and heterogeneity of the outcomes in the study.

## 4. Discussion

### 4.1. Findings

We identified nine RCTs investigating the influence of CBT on depressive symptoms and sleep quality in menopausal women, encompassing a total of 923 participants across three different countries. Our meta-analysis indicated a statistically significant large-sized effect in alleviating depressive symptoms and a considerable medium effect in improving sleep quality for menopausal women. Most trials, as assessed with a risk of bias evaluation, were rated as having moderate overall quality, with low statistical heterogeneity for depressive symptoms and high heterogeneity for sleep quality.

Concerning intervention specifics, integrated cognitive-behavioral interventions typically span a treatment duration of approximately 4 to 8 weeks. In our investigation, we observed an average application frequency for cognitive-behavioral interventions at 5.5 weeks, coupled with an average session count of 5.5 and an average session duration of 60 min. This is in contrast to a prior meta-analysis study that focused on adults aged 18 to under 65 [27], where cognitive-behavioral interventions demonstrated an average frequency of 5.4 weeks, an average session count of 5.5, and an average session duration of 90 min. Although the average intervention frequencies and counts were quite similar between our review of the literature and the previous one, the average intervention duration in the RCTs that were included in our review was notably shorter. This difference may be attributed to the fact that our study primarily employed face-to-face education, while the previous study utilized a variety of educational mediums, including face-to-face instruction, telephone sessions, and audio internet-based self-help programs, demonstrating its effectiveness through diverse educational channels.

Since there were a limited number of included RCTs, our ability to effectively assess the presence of publication bias was not possible. It is recommended for meta-analyses with ten or more studies to use tests such as funnel plot asymmetry and Egger’s regression; given the small number of trials in our analysis, it lacked statistical power, potentially indicating reporting bias [26]. Additionally, our search was limited to English-language studies, possibly leading to the oversight of relevant research in other languages meeting the review’s criteria. The exclusion of gray literature might have also increased the likelihood of publication bias.

Moreover, the use of various comparison conditions in the studies might have influenced the findings. The two RCTs included in the quantitative analysis (meta-analysis) reported a significant reduction in depressive symptoms, while the control groups, such as the alternate group education and usual care groups, did not show an intervention effect. This suggests the possibility that non-specific therapeutic factors, such as providing care and social support, rather than the intervention itself, contributed to the reduction in depressive symptoms.

In our meta-analysis, two studies indicated that CBT-based interventions effectively reduced depressive symptoms in middle-aged individuals, including those experiencing the menopausal phase. These results align with previous meta-analyses on the impact of CBT on depressive symptoms in women with polycystic ovarian syndrome (PCOS) [28]. Nevertheless, additional research studies are required in the future to establish conclusive findings regarding the impact of CBT on depressive symptoms in climacteric women.

Regarding sleep quality, the analysis indicated a 1.44% improvement from baseline to follow-up in individuals who underwent CBT, whereas the control group, without CBT, exhibited only a 0.05% decrease from baseline. These results align with previous meta-analyses focused on the impact of CBT on enhancing sleep quality in menopausal women [29]. Cognitive-behavioral techniques, especially those rooted in CBT, were recognized as more effective in improving sleep quality compared to alternative approaches for addressing sleep problems.

Our findings have unveiled that, in eight out of nine studies, CBT was predominantly delivered via face-to-face sessions. Nevertheless, individuals grappling with depressive symptoms often exhibit a deficiency in motivation to partake in such in-person programs. Furthermore, depressed menopausal women frequently find themselves lacking essential social support [30]. To enhance the accessibility of CBT, we propose considering alternative avenues, specifically online delivery methods, which include standalone applications and web-based platforms or a hybrid approach incorporating both face-to-face and online application delivery. The advantage of utilizing applications lies in their capacity to be accessed from virtually any location with an internet connection. This accessibility extends a lifeline to participants, particularly those residing in remote areas or facing mobility constraints. Additionally, online CBT empowers participants to engage in therapy at their convenience, accommodating individuals with hectic schedules or those encountering difficulty in attending traditional in-person sessions regularly. Recognizing the evolving landscape of education and support, we emphasize the importance of merging online and offline approaches that align with contemporary trends. This holistic strategy fosters a sense of community, providing a platform for individuals to come together, cultivate social support networks, and amplify their social connections.

In the realm of menopausal women’s well-being, it becomes evident that they are frequently entangled in a web of physical symptoms and depressive states [4,5]. This highlights the vital need for all-encompassing educational interventions, integrating CBT and tailored education addressing the physical aspects of the menopausal transition. Given the intricate interplay between physiological changes and psychological distress, a research-driven approach becomes imperative to craft effective strategies that optimize well-being during menopause. Therefore, it is deemed necessary to develop and implement more comprehensive programs that consider lifestyle, chronic disease management, and psychological support for middle-aged women.

### 4.2. Limitations

Our review has several limitations to address. Firstly, the blinding methods were either inadequately described or unclear in the majority (*n* = 5) of the included studies, potentially introducing attrition bias [26]. Secondly, there was variability in intervention methods among the studies in our meta-analysis, despite all being rooted in CBT. Additional constraints included small sample sizes in some RCTs (specifically, five out of the twelve studies had fewer than 90 participants in total) and the potential presence of publication bias. Thirdly, in relation to insomnia and CBT, the common reason insomnia patients seek medical attention is believed to be a sense of helplessness in regulating sleep rather than the insomnia itself. Therefore, to enhance the effectiveness of CBT, it is crucial to facilitate direct patient involvement in the treatment process. Exploring reasons for resistance during the treatment and finding appropriate compromises are necessary, even when patients exhibit resistance. For sleep-related issues, Integrated Cognitive-Behavioral Interventions, combining stimulus control therapy, sleep restriction therapy, sleep hygiene education, and cognitive restructuring, are preferred. In terms of methodology, it is essential to recognize the challenges in conducting face-to-face education due to the inherent difficulties in psychosocial interventions, making various media essential for efficient education. Additionally, considering the expertise of intervention providers is crucial, indicating the need for an integrated cognitive-behavioral intervention that factors in these elements.

Lastly, the results of our review of the literature provide evidence for the effectiveness of cognitive-behavioral interventions in alleviating insomnia and depression. However, a limitation is the absence of studies evaluating the efficacy of sleep using objective tools such as actigraphy or polysomnography; the assessment relied solely on self-reported sleep diaries or questionnaires. For insomnia, which can be exacerbated by misconceptions and habits related to sleep, it is considered essential in the realm of health intervention to establish controllable behavioral goals through integrated interventions such as cognitive-behavioral interventions. This involves setting achievable behavioral goals, correcting misconceptions, and exploring behavioral approaches.

## 5. Conclusions

In summary, when it comes to CBT for climacteric women, we found that it did have a statistically significant impact on reducing depressive symptoms, and the effect size observed was large. Also, for sleep-related problems, especially sleep quality, we did observe a large effect size with statistically significant results. The overall variability among the trials was low. As our findings suggest, research concerning CBT for climacteric women is still in its early stages. Our review underscores the necessity for studies on CBT for climacteric women, especially outcomes of depressive symptoms and sleep quality to encompass a broader geographical scope including more countries, to be conducted on a larger scale to yield more robust evidence. In addition, caution is advised when interpreting these findings because of variations in methodology and the overall quality of the studies. Further clinical trials are required to determine the optimal number of CBT sessions required to effectively treat depression in climacteric women.

## Figures and Tables

**Table 1:** Descriptive summary of included studies (*N* = 9).

Study	Sample Size (*N*)	Age (Mean + SD)	Intervention 1. Mode of Therapy 2. Duration/No. of Sessions 3. Min/Session 4. Provider	Control	Follow-Up Times	Outcomes (Scale)
Abdelaziz et al. (2022) [17] Saudi Arabia	80	53.06 ± 4.28	1. Group (online) 2. 6 online modules 3. 20–30 min 4. Researchers	Usual care	6 weeks	Primary variables: PSQI ISI Secondary variables: Sleep diary (SOL, frequency of nighttime awakenings, sleep quality, TIB, TST, and sleep efficiency)
Drake et al. (2019) [18] USA	150	56.44 ± 5.64	1. Individual (in person) 2. 6 weekly sessions 3. Unclear 4. Clinical psychologist	Minimal intervention	2 weeks post-treatment and at 6 months	ISI, FORD, TST, SOL, nighttime awakenings, waking after sleep, and sleep efficiency
Green et al. (2019) [19] Canada	90	Intervention 53.27 ± 3.69 Control 52.88 ± 4.39	1. Group (in person) 2. 12 weekly sessions 3. 120 4. Psychologists and graduate-level trainees	Wait-list	12 weeks post-baseline, and at 3 months post- treatment	BDI-II, MADRS, PSQI, HAM, FSFI, and GCS-sex
Ham et al. (2020) [20] South Korea	44	Intervention 53.83 ± 6.64 Control 55.45 ± 4.43	1. Group and individual 2. Weekly sessions over 4 weeks 3. 30 to 60 min 4. Psychologist and psychiatrist	Group education	1 month and 12 months	CES-D, PSQI, ISI, and MenQoL
Hardy et al. (2018) [21] UK	124	54.09 ± 3.4	1. Individual 2. Self-help Cognitive Behavioral Therapy over 4 weeks 3. Unclear 4. Self-help	No treatment waitlist control	6 and 20 weeks post-randomization	PSQI, HFRS, HF frequency, HFNS beliefs and behaviors, MRQ, WHO, and WSAS
Kalmbach et al. (2019) [22] USA	117	56.34 ± 5.41	1. Group 2. 6 face-to-face sleep therapy sessions 3. Unclear 4. Registered nurse	Sleep hygiene education	6 months	BDI-II, DBAS, PSAS Cognitive, ERRI, PSWQ, and PSAS Somatic
Kalmbach et al. (2019) [23] USA	150	56.44 ± 5.64	1. Individual (in person) 2. 6 weekly sessions 3. Unclear 4. Registered nurse	Sleep hygiene education	Post-treatment and at 6 months	ESS, diary-based daytime sleepiness and FSS
McCurry et al. (2016) [24] USA	88	Intervention 55.0 ± 3.5 Control 54.7 ± 4.7	1. Individual (in person) 2. 6 CBTs in 8 weeks 3. 20–30 min 4. CBT experts (coaches)	Menopause education control (phone session)	8 and 24 weeks post-randomization	ISI, PSQI, sleep diary, diary of wake time after sleep onset, diary of total sleep time, and diary of sleep efficiency
Reddy et al. (2019) [25] India	80	Intervention 48.63 ± 0.55 Control 49.16 ± 0.8	1. Individual (in person) 2. 6 weekly group CBT sessions 3. 50–60 min 4. Primary provider	TAU	1 month and 6 months	CES-D

Abbreviations: PSQI—Pittsburgh Sleep Quality Index; CES-D—Center for Epidemiologic Studies Depression Scale; BDI-II—Beck Depression Inventory-II; ESS—The Epworth Sleepiness Scale; MADRS—Montgomery–Asberg Depression Rating Scale; TAU—treatment as usual; FORD—Ford Insomnia Response to Stress Test; SOL—sleep onset latency; TIB—time between bedtime and waking time; TST—total sleep time; HAM—Hamilton Anxiety Measure; FSFI—Female Sexual Function Inventory; GCS-sex—Sexual concerns item of the Greene Climacteric Scale; MenQoL—Menopausal Quality of Life; HFRS—hot flush rating scale; MRQ—Menopause Representation Questionnaire; WHO—Women’s Health Questionnaire; WSAS—Work and Social Adjustment Scale; DBAS—Dysfunctional Beliefs and Attitudes; PSAS Cognitive—pre-sleep arousal scale cognitive factor; ERRI—event-related rumination inventory; PSWQ—Penn State worry questionnaire.

## Data Availability

The data presented in this study are available upon request from the corresponding author. The data are not publicly available because of respondents’ privacy.
